# *Clostridium difficile* Ribotype 027, Toxinotype III, the Netherlands

**DOI:** 10.3201/eid1205.051350

**Published:** 2006-05

**Authors:** Ed J. Kuijper, Renate J. van den Berg, Sylvia Debast, Caroline E. Visser, Dick Veenendaal, Annet Troelstra, Tjallie van der Kooi, Susan van den Hof, Daan W. Notermans

**Affiliations:** * Leiden University Medical Center, Leiden, the Netherlands;; † St Jansdal Hospital, Harderwijk, the Netherlands;; ‡ Academic Medical Center, Amsterdam, the Netherlands;; § The Public Health Laboratory, Haarlem, the Netherlands;; ¶ Utrecht Medical Center, Utrecht, the Netherlands;; # National Institute for Public Health and the Environment (RIVM), Bilthoven, the Netherlands

**Keywords:** Clostridium difficile, PCR ribotype 027, toxinotype IIII, epidemic, Harderwijk, dispatch

## Abstract

Outbreaks due to *Clostridium difficile* polymerase chain reaction (PCR) ribotype 027, toxinotype III, were detected in 7 hospitals in the Netherlands from April 2005 to February 2006. One hospital experienced at the same time a second outbreak due to a toxin A–negative *C. difficile* PCR ribotype 017 toxinotype VIII strain. The outbreaks are difficult to control.

Since March 2003, outbreaks of severe cases of *Clostridium difficile*–associated disease (CDAD) were reported in hospitals in Montreal and Quebec (*1**,**2*). Increased virulence was suspected, since the proportion of patients with CDAD who died within 30 days after diagnosis rose from 4.7% in 1991–1992 to 13.8% in 2003 (*1*). In addition, the Centers for Disease Control and Prevention reported a growing threat of CDAD in US hospitals and found the strain to be associated with high illness and death rates during hospital outbreaks in 11 states (*3*). The increased virulence was considered to be associated with the production of a binary toxin and an increased production of toxins A and B (*4*). Further characterization of this strain showed that it belonged to toxinotype III, pulsed-field gel electrophoresis (PFGE) type NAP1, restriction endonuclease analysis group BI, and polymerase chain reaction (PCR) ribotype 027 (*2**,**3*). Toxinotyping involves detecting polymorphisms in the toxin A and B and surrounding regulatory genes, an area of the genome known collectively as the pathogenicity locus or PaLoc (*5*). By toxinotyping, 24 different types can be recognized, whereas the library of PCR ribotypes comprises 116 distinct types of *C. difficile* identified on the basis of differences in amplification profiles generated (*6*). The PCR ribotype 027, toxinotype III, strain is resistant to ciprofloxacin and the newer generation of fluoroquinolones, such as gatifloxacin, levofloxacin, and moxifloxacin (*3*). Exposure of patients to fluoroquinolones and cephalosporins is recognized as a risk factor for CDAD caused by 027 (*2**,**3*). Increasing use of fluoroquinolones in US healthcare facilities may have provided a selective advantage for this epidemic strain and promoted its widespread emergence.

## The Outbreaks

In July 2005, the medical microbiologic laboratory at the Leiden University Medical Center was requested to type *C. difficile* strains from an outbreak in a hospital (hospital l) in Harderwijk (Figure, Table). The incidence of CDAD in the hospital had increased from 4 per 10,000 patient admissions in 2004 to 83 per 10,000 admissions from April through July 2005. Cultured isolates were subsequently identified as toxinotype III and PCR ribotype 027 (*7*). The strain also had the binary toxin genes and contained an 18-bp deletion in a toxin regulator gene (*tcdC*). As determined by E test (AB Biodisk, Solna Sweden), the isolates were resistant to erythromycin (MIC >256 mg/L) and ciprofloxacin (MIC >32 mg/L) and susceptible to clindamycin (MIC 2 mg/L) and metronidazole (MIC 0.19 mg/mL). Measures taken by the hospital included isolating all patients with diarrhea until 2 tests were negative for *C. difficile* toxin, cohorting all *C. difficile*–infected patients on a separate ward, banning all fluoroquinolone use, and limiting use of cephalosporins and clindamycin. A case-control study is being performed in the hospital to determine risk factors for acquiring this strain, and a follow-up study will determine the rate of complications and relapses. As of January 2006, the situation appears to be under control since the number of patients per month with positive test results has decreased. All 9 CDAD cases from September 2005 to January 2006 were caused by non-027 ribotypes. Therefore, cohort isolation and the limitation on antimicrobial agents have been stopped.

**Figure:**
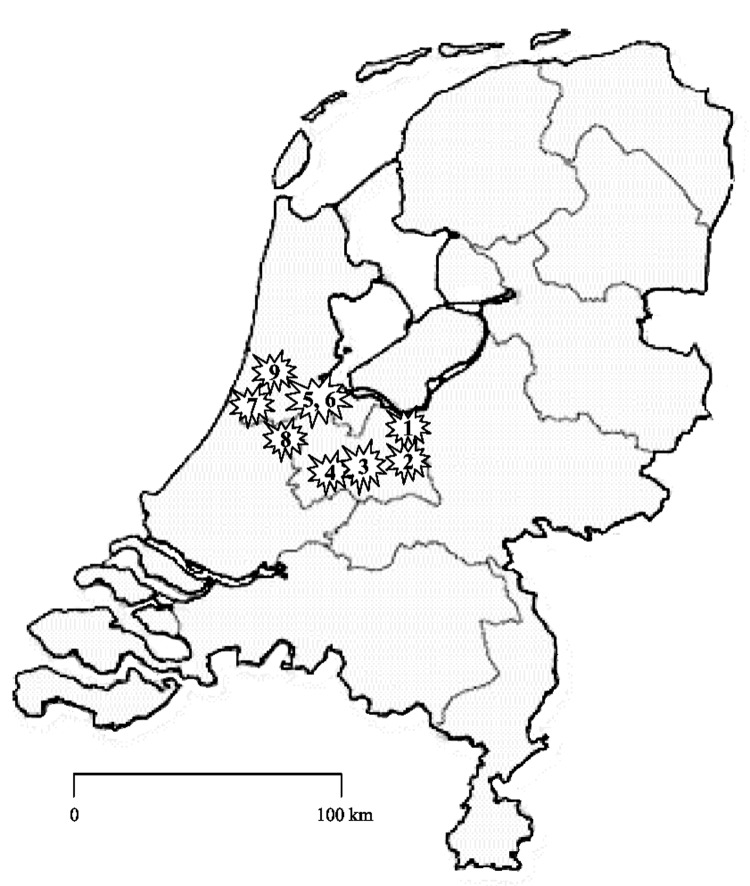
Location of the hospitals with outbreaks of *Clostridium difficile*–associated diarrhea in the Netherlands. The numbers correspond with those in the Table.

**Table:** Characteristics of 9 hospitals with patients with *Clostridium difficile*–associated diarrhea due to PCR ribotype 027, toxinotype III*

Hospital no. and setting	No. beds	Admissions						
		Incidence/ 10,000, before outbreak	Maximum incidence/ mo/10,000, during outbreak†	Date of outbreak onset	Total no. CDAD patients in given period, 2005	Deaths, 30 d	No. strains studied	No. toxinotype III, PCR ribotype 027 strains
1. Harderwijk	341	4	83	Apr 2005	51, Apr–Nov	3	30	19
2. Amersfoort	600	11	87	May 2005	85, Jan–Dec	19	50	15
3. Utrecht	1,013	16	–	No outbreak	37, Jun–Dec	Unk.	17	6
4. Nieuwegein	584	11	–	No outbreak	13, Jan–Dec	Unk.	4	1
5. Amsterdam	1,002	38	52	June 2005	68, Jan–Oct	1	28	12
6. Amsterdam	310	10	66	Apr–May 2005	42, Jan–Oct	Unk.	34	16
7. Haarlem	744	7	27	2004	66, Jan–Dec	Unk.	9	7
8. Hoofddorp	455	3	76	Jan 2005	73, Jan–Dec	Unk.	8	8
9. Beverwijk	383	4	47	2002	24, Jan–Dec	Unk.	4	3

*PCR, polymerase chain reaction; CDAD, *Clostridium difficile*–associated diarrhea; unk., unknown. †Timeframe 2–4 mo.

A second epidemic occurred in another hospital 30 km from the first hospital (hospital 2, Amersfoort) and was probably related to the outbreak in hospital 1 through a transferred patient with CDAD. Isolates obtained from patients were indistinguishable from the Harderwijk isolates. After the index patient was transferred, the incidence of CDAD, which had been 2–3 cases per month for the last 2 years, rose to an average of 15 cases per month during May, June, and July. From August to December, the number of CDAD patients per month was 7, 7, 8, 14, and 10, respectively. Of the 85 CDAD patients found through December 2005, 19 (22%) patients died, and 16 (19%) had relapses. Of 50 strains characterized at the reference laboratory, 15 belonged to PCR ribotype 027, and 14 belonged to PCR ribotype 017, toxinotype VIII. The 017 strain had a deletion of the toxin A gene, did not contain genes for binary toxin production, and had a normal *tcdC* gene.

In response to the outbreaks in the Netherlands, the Centre for Infectious Disease Control at the National Institute for Public Health and the Environment in Bilthoven organized a meeting with experts in the fields of microbiology, infectious diseases, infection control, and epidemiology. The team agreed to combine parts of existing national hospital guidelines relevant for infection control of CDAD and to use national and international experience in drawing up specific CDAD guidelines for infection control and treatment separate for hospitals and nursing homes. Diagnostic facilities were increased and made accessible for all microbiology laboratories in the Netherlands. Relevant professionals were informed through different communication channels, including various scientific societies (*7*). Plans were made to register and monitor new outbreaks. Laboratories were encouraged to send patient isolates or fecal samples for typing to the reference laboratory in Leiden when an outbreak was suspected on the basis of an increase in monthly incidence or a rapid spread of clinically suspected cases.

Subsequently, 3 hospitals in the western part of the country (hospitals 7–9) also reported an increase in incidence of severe CDAD. In 2005, the public health laboratory serving these 3 hospitals diagnosed CDAD in 163 patients. Of 21 strains sent to the reference laboratory, 18 were identified as PCR ribotype 027, toxinotype III (Table). Retrospectively, an increase of CDAD was first evident in July 2004 for hospital 7 and in 2002 for hospital 9. The public health laboratory diagnosed CDAD in 120 patients in 2004, in 58 in 2003, and in 47 in 2002. No strains or fecal samples before 2005 were available for typing. A nursing home in the same region was also found to have patients with CDAD due to PCR ribotype 027, with evidence of spread within the facility. No epidemiologic relationship could be established between this region ad that of the first 2 outbreaks.

Two hospitals in the center of the Netherlands (hospitals 3 and 4) did not notice an increase in the incidence of patients with CDAD but submitted strains to the reference laboratory for typing. Type 027 was found in 6 (35%) of 17 and 1 (25%) of 4 isolates tested, respectively. None of the 7 patients with CDAD due to type 027 had severe disease.

A cluster of 12 patients with CDAD by PCR ribotype 027, toxinotype III, was reported in July and August in a large teaching hospital in Amsterdam (hospital 5). One patient died from consequences of CDAD, and severe complications developed in 2 other patients. Another hospital in Amsterdam (hospital 6) also reported an increase of severe cases of CDAD in July 2005 in geriatric patients. Strains cultured from fecal samples of 7 patients in August 2005 showed PCR ribotype 027, toxinotype III.

## Conclusions

Shortly after the reports in June 2005 of the detection of *C. difficile* PCR ribotype 027, toxinotype III, in English hospitals, this more virulent type was detected in the Netherlands (*7**,**8*). More recently, the reference laboratory at Leiden University Medical Center also detected this strain in samples from Belgium as a causative agent of outbreaks of CDAD (*9*). The virulence factors of this emerging strain are not well understood. It contains a binary toxin, but the importance of binary toxin as a virulence factor in *C. difficile* has not been established. The binary toxin, an actin-specific adenosine diphosphate–ribosyltransferase, is encoded by the *cdtA* gene (the enzymatic component) and the *cdtB* gene (the binding component), which are not located within the pathogenicity locus (*10**,**11*). Nonpathogenic strains that contain *cdtA* and *cdtB* genes but lack the pathogenicity locus are also capable of producing binary toxin. The binary toxin is present in ≈6% of all *C. difficile* isolates, irrespective of the toxinotype (*10**,**11*). We therefore consider it likely that the binary toxin in PCR ribotype 027, toxinotype III, strains merely reflects clonal spread of a restricted number of strains.

The importance of the 18-bp deletion in *tcdC* of the PCR ribotype 027, toxinotype III, strains is also unknown. *tcdC* is considered a negative regulator of the production of toxins A and B, but whether this 18-bp deletion results in a nonfunctional product is unknown (*3*). A recent report, however, indicates that toxinotype III isolates produce toxins A and B in considerably greater quantities in vitro than toxinotype 0 isolates (*4*). On the other hand, deletions in *tcdC* are frequently present in toxinogenic isolates. Of 32 toxinogenic strains studied in 2002, 8 belonged to toxinotypes 0, V, and VI and contained deletions in *tcdC* of 18 bp or 39 bp, although this deletion was not associated with severity of disease (*12*).

The PCR ribotype 027, toxinotype III, strain has a characteristic antimicrobial susceptibility pattern, since it is resistant to the newer fluoroquinolones and erythromycin but susceptible to clindamycin. Macrolide, lincosamide, and streptogramin B (MLSB) resistance is usually due to an *erm*(*B*) gene, but PCR ribotype 027 and toxinotype III strain did not contain an *erm*(*B*) gene. All current PCR ribotype 027 and toxinotype III strains but no historical isolates (obtained before 2001) were resistant to gatifloxacin and moxifloxacin (*3*). The resistance for ciprofloxacin and newer fluoroquinolones is not specific for the new virulent strains, since it has also been found in other common PCR ribotypes in the United Kingdom (*13*).

The observation that outbreaks due to different strains can occur simultaneously emphasizes that microbiologic monitoring is important for epidemiologic studies of CDAD. PCR ribotype 017 strain lacks a part of the toxin A gene and was first recognized as a cause of an outbreak in Canada in 1999 (*14*). Subsequently, toxin A–negative, toxin B–positive strains caused outbreaks of CDAD in Ireland (D. Drudy, pers. comm.), Argentina (M.C. Legaria, et al., unpub. data), and the Netherlands (*15*).

The outbreaks in the Netherlands are difficult to control. In the Harderwijk epidemic, using rapid diagnostic tests for CDAD and cohort isolation in combination with restricting use of fluoroquinolones and cephalosporins appeared to be successful. Outbreaks in the other hospitals are still not completely under control.
